# New Coleoptera records from New Brunswick, Canada: Sphindidae, Erotylidae, Monotomidae, and Cryptophagidae

**DOI:** 10.3897/zookeys.179.2466

**Published:** 2012-04-04

**Authors:** Reginald P. Webster, Jon D. Sweeney, Ian DeMerchant

**Affiliations:** 1 Natural Resources Canada, Canadian Forest Service - Atlantic Forestry Centre, 1350 Regent St., P.O. Box 4000, Fredericton, NB, Canada E3B 5P7

**Keywords:** Sphindidae, Erotylidae, Monotomidae, Cryptophagidae, new records, Canada, New Brunswick

## Abstract

Two species of Sphindidae, *Odontosphindus denticollis* LeConteand *Sphindus trinifer* Casey, are reported for the first time for New Brunswick. Another species, *Sphindus* near *americanus* LeConte is reported from the province but may be an undescribed species, pending further study. Five species of Erotylidae are newly recorded for the province, including *Tritoma humeralis* Fabricius and *Tritoma sanguinipennis* (Say), which are new to the Maritime provinces. Three species of Monotomidae are added to the New Brunswick faunal list, including *Pycnotomina cavicollis* (Horn), which is newly recorded for the Maritime provinces. Six additional species of Cryptophagidae are reported for the province and the presence of *Antherophagus convexulus* LeContein New Brunswick is confirmed. *Cryptophagus pilosus* Gyllenhal and *Myrmedophila americana* (LeConte) are newly reported to the Maritime provinces.

## Introduction

The Sphindidae, Erotylidae, and Monotomidae of the Maritime provinces (New Brunswick, Nova Scotia, Prince Edward Island) were reviewed by Majka (2007, 2010) and Majka and Bousquet (2010), respectively. The Cryptophagidae of Atlantic Canada were reviewed by Majka et al. (2010a) (Atomariinae) and Majka and Langor (2010) (Cryptophaginae). Intensive collecting in New Brunswick by the first author since 2003 and records obtained more recently from by-catch samples during a study to develop improved lures for the detection of invasive species of Cerambycidae have yielded additional new provincial records in the above families. The purpose of this paper is to report on these new records. A brief synopsis of each family is included in the results below.

## Methods and conventions

The following records are based on specimens collected during a general survey by the first author to document the Coleoptera fauna of New Brunswick and from by-catch samples obtained from trapping experiments conducted to develop tools for the detection of invasive species of Cerambycidae.

### Collection methods

Various collection methods were employed to collect the species reported in this study. Details are outlined in Campbell (1973) and Webster et al. (2009, Appendix). See Webster et al. (in press) for details of the methods used for deployment of Lindgren 12-funnel traps and sample collection. A description of the habitat was recorded for all specimens collected during this survey. Locality and habitat data are presented exactly as on labels for each record. This information, as well as additional collecting notes, is summarized and discussed in collection and habitat data section for each species.

### Distribution

Distribution maps, created using ArcMap and ArcGIS, are presented for each species in New Brunswick. Every species is cited with current distribution in Canada and Alaska, using abbreviations for the state, provinces, and territories. New records for New Brunswick are indicated in bold under Distribution in Canada and Alaska. The following abbreviations are used in the text:

**Table:** 

**AK**	Alaska	**MB**	Manitoba
**YT**	Yukon Territory	**ON**	Ontario
**NT**	Northwest Territories	**QC**	Quebec
**NU**	Nunavut	**NB**	New Brunswick
**BC**	British Columbia	**PE**	Prince Edward Island
**AB**	Alberta	**NS**	Nova Scotia
**SK**	Saskatchewan	**NF & LB**	Newfoundland and Labrador

Acronyms of collections examined or where specimens reside referred to in this study are as follows:

AFCNatural Resources Canada, Canadian Forest Service - Atlantic Forestry Centre, Fredericton, New Brunswick, Canada

CNCCanadian National Collection of Insects, Arachnids and Nematodes, Agriculture and Agri-Food Canada, Ottawa, Ontario, Canada

NBMNew Brunswick Museum, Saint John, New Brunswick, Canada

RWCReginald P. Webster Collection, Charters Settlement, New Brunswick, Canada

## Results

### Species accounts

All records are species newly recorded for New Brunswick, Canada unless noted otherwise (additional records). Species followed by ** are newly recorded from the Maritime provinces of Canada.

The classification of the Sphindidae, Erotylidae, Monotomidae, and Cryptophagidae follows Bouchard et al. (2011).

**Table 1.** Species of Sphindidae, Erotylidae, Monotomidae, and Cryptophagidae recorded from New Brunswick.

**Family Sphindidae Jacquelin du Val**
**Subfamily Odontosphindinae Sen Gupta and Crowson**
*Odontosphindus denticollis* LeConte*
**Subfamily Sphindinae** **Jacquelin du Val**
*Sphindus* near *americanus* LeConte
*Sphindus trinifer* Casey*
*Eurysphindus hirtus* LeConte
**Family Erotylidae Latreille**
**Subfamily Languriinae Hope**
**Tribe Languriini Hope**
*Acropteroxys gracilis* (Newman)
**Subfamily Erotylinae Latreille**
**Tribe Dacnini Gistel**
*Dacne quadrimaculata* (Say)*
**Tribe Tritomini Curtis**
*Triplax dissimulator* (Crotch)
*Triplax frosti* Casey
*Triplax macra* LeConte*
*Triplax thoracica* Say
*Tritoma humeralis* Fabricius*
*Tritoma pulchra* Say*
*Tritoma sanguinipennis* (Say)**
**Family Monotomidae Laporte**
**Subfamily Rhizophaginae Laporte**
*Rhizophagus brunneus brunneus* Horn
*Rhizophagus dimidiatus* Mannerheim
*Rhizophagus minutus rotundicollis* Bousquet*
*Rhizophagus remotus* LeConte*
**Subfamily Monotominae Laporte**
*Monotoma bicolor* Villa and Villa
*Monotoma longicollis* (Gyllenhal)
*Monotoma picipes* Herbst
*Monotoma producta* LeConte
*Pycnotomina cavicollis* (Horn)**
**Tribe Cryptophagini Kirby**
**Family Cryptophagidae Kirby**
**Subfamily Cryptophaginae Kirby**
*Antherophagus convexulus* LeConte
*Antherophagus ochraceus* Melshiemer
*Cryptophagus acutangulus* Gyllenhal*
*Cryptophagus fallax* Balfour-Browne
*Cryptophagus mainensis* Casey*
*Cryptophagus pilosus* Gyllenhal**
*Henoticus serratus* (Gyllenhal)*
*Henotiderus centromaculatus* Reitter*
*Pteryngium crenatum* (Fabricius)*
*Telmatophilus americanus* LeConte
*Telmatophilus typhae* (Fallen)
**Tribe Atomeriini LeConte**
**Subfamily Atomeriinae LeConte**
*Atomaria (Anchicera) apicalis* Erichson
*Atomaria (Anchicera) distincta* Casey
*Atomaria (Anchicera) ephippiata* Zimmerman
*Atomaria (Anchicera) fuscata* Schonherr
*Atomaria (Anchicera) lewisi* Reitter
*Atomaria (Anchicera) pusilla* (Paykull)
*Atomaria (Anchicera) * Stephens

Notes. *New to province, **New to Maritime provinces.

#### Family Sphindidae Jacquelin du Val, 1860

The Sphindidae (cryptic slime mold beetles) live in or on slime-mold sporocarps, and both larvae and adults feed on spores and supporting structures of the slime molds (McHugh 2002). Campbell (1991a) reported five species from Canada but none from New Brunswick and the other Maritime provinces, although Lafontaine et al. (1987) reported *Odontosphindus denticollis* LeConte from the Cape Breton Highlands National Park in Nova Scotia. Majka (2010) reported *Sphindus americanus* LeConte and *Eurysphindus hirtus* LeConte from New Brunswick. However, the identification of *Sphindus americanus* was considered provisional due to the poor quality of the specimen. *Sphindus americanus* and *Eurysphindus hirtus* were newly reported from Nova Scotia (Dollin et al. 2008; Majka 2010). Here, we report *Sphindus trinifer* Casey and *Odontosphindus denticollis* LeConte for the first time for the province. Another species, *Sphindus* near *americanus* LeConte is reported from the province but may be an undescribed species, pending further study and additional specimens. This is presumably the same species reported as *Sphindus americanus* by Majka (2010). A list of the species currently known from New Brunswick is given in Table 1.

##### Subfamily Odontosphindinae Sen Gupta and Crowson, 1979

###### Odontosphindus denticollis

LeConte, 1878

http://species-id.net/wiki/Odontosphindus_denticollis

Map 1

####### Material examined.

**New Brunswick, Carleton Co.**, Meduxnekeag Valley Nature Preserve, 46.1907°N, 67.6740°W, 20.VI.2009, R. P. Webster, mixed forest on slime mould (*Stemontis* sp.) on rotted log (5, RWC). **Queens Co.**, Cranberry Lake P.N.A. (Protected Natural Area), 46.1125°N, 65.6075°W, 11–18.VI.2009, 18–25.VI.2009, R. Webster & M.-A. Giguère, old red oak forest, Lindgren funnel traps (2, AFC, RWC); same locality data and forest type but 13–25.V.2011, M. Roy & V. Webster, Lindgren funnel trap (1, RWC). **York Co.**, Charters Settlement, 45.8395°N, 66.7391°W, 18.VII.2006, R. P. Webster, mixed forest, on slime mould (*Stemontis* sp.) on rotted log (1, RWC); 15 km W of Tracy off Rt. 645, 45.6848°N, 66.8821°W, 28.VI–7.VII.2009, R. Webster & M.-A. Giguère, old red pine forest, Lindgren funnel traps (2, AFC, RWC).

####### Collection and habitat data. 

This species was reported on the slime mold, *Fuligo septica* (L.) Wigg. by Lawrence and Newton (1980). In New Brunswick, adults were collected from *Stemontis* species (slime mold) on rotted logs in mixed forests and from Lindgren funnel traps deployed in an old red oak (*Quercus rubra* L.) forest and an old red pine (*Pinus resinosa* Ait.) forest. Adults were collected during May, June, and July.

####### Distribution in Canada and Alaska.

ON, QC, **NB**, NS (Lafontaine et al. 1987; Campbell 1991a; Dollin et al. 2008; Bishop et al. 2009; Majka 2010).

**Map 1.**
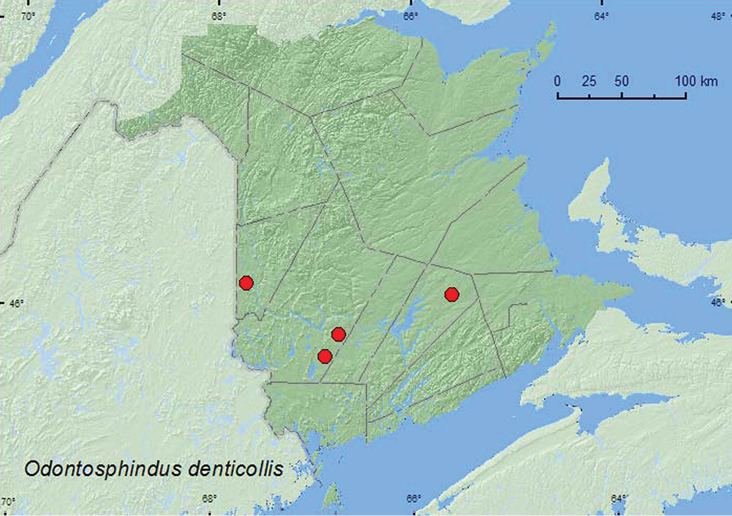
Collection localities in New Brunswick, Canada of *Odontosphindus denticollis*.

##### Subfamily Sphindinae Jacquelin du Val, 1860

###### Sphindus species near americanus

LeConte, 1866

Map 2

####### Material examined.

**New Brunswick, York Co.**, Charters Settlement, 45.8395°N, 66.7391°W, 26.VIII.2007, R. P. Webster, mixed forest, u.v. light (1, RWC); 15 km W of Tracy off Rt. 645, 45.6848°N, 66.8821°W, 15–21.VI.2009, R. Webster & M.-A. Giguère, old red pine forest, Lindgren funnel traps (2, AFC, RWC).

####### Collection and habitat data.

This species was collected at an ultraviolet light near a mixed forest and captured in Lindgren funnel traps deployed in an old red pine forest. Adults were captured during June and August.

####### Distribution in Canada and Alaska.

Majka (2010) considered the identification of *Sphindus americanus* as provisional for New Brunswick due to the poor condition of the specimen. The above specimens are similar to *Sphindus americanus* in possessing a two-segmented antennal club, but differ in other characters from specimens of *Sphindus americanus* in the C.N.C. and may be an undescribed species (Serge Laplante, personal communication). In Canada, *Sphindus americanus* was reported from British Columbia, Alberta, Ontario, and Quebec by Campbell (1991a). *Sphindus americanus* was first reported from Nova Scotia by Dollin et al. (2008), and Majka (2010) considered this species common and widespread in the province (but see below).

**Map 2.**
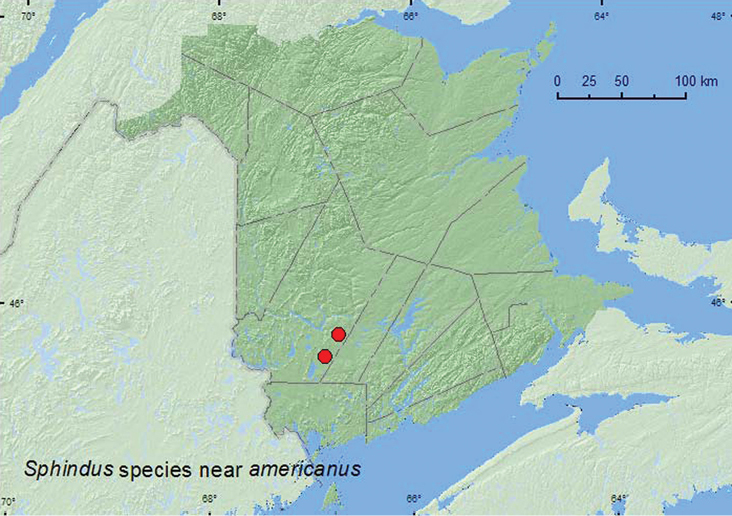
Collection localities in New Brunswick, Canada of *Sphindus* near *americanus*.

###### Sphindus trinifer

Casey, 1898**

http://species-id.net/wiki/Sphindus_trinifer

Map 3

####### Material examined.

**New Brunswick, Carleton Co.**, Jackson Falls, Bell Forest, 46.2200°N, 67.7231°W, 26.VI.2007, 25.VII.2007, R. P. Webster, mature hardwood forest, u.v. light (2, NBM, RWC); same locality and forest type, 4–12.VI.2008, R. P. Webster, Lindgren funnel trap (1, RWC); same locality and habitat data but 9–14.V.2009, 14–20.V.2009, 8–16.VI.2009, 16–21.VI.2009, Webster & M.-A. Giguère, Lindgren funnel traps (4, AFC, RWC). **Charlotte Co.**, 10 km NW of New River Beach, 45.2110°N, 66.6170°W, 16–26.VII.2010, R. Webster & C. MacKay, old growth eastern white cedar forest, Lindgren funnel trap (1, AFC). **Queens Co.**, Cranberry Lake P.N.A., 46.1125°N, 65.6075°W, 21–27.V.2009, 5–11.VI.2009, R. Webster & M.-A. Giguère, old red oak forest, Lindgren funnel traps (5, AFC); Grand Lake Meadows P.N.A., 45.8227°N, 66.1209°W, 19–31.V.2010, R. Webster & C. MacKay, old silver maple forest with green ash and seasonally flooded marsh, Lindgren funnel trap (1, AFC); same locality data and forest type, 21.VI-5.VII.2011, M. Roy & V. Webster, Lindgren funnel trap (1, NBM). **Restigouche Co.**, Dionne Brook P.N.A., 47.9030°N, 68.3503°W, 30.V-15.VI.2011, 9–23.VIII.2011, M. Roy & V. Webster, old-growth northern hardwood forest, Lindgren funnel traps (3, NBM, RWC). **Sunbury Co.**, Acadia Research Forest, 45.9866°N, 66.3841°W, 19–25.V.2009, 25.V-2.VI.2009, 24–30.VI.2009, R. Webster & M.-A. Giguère, mature (110-year-old) red spruce forest with scattered red maple and balsam fir, Lindgren funnel traps (6, AFC). **York Co.**, Charters Settlement, 45.8395°N, 66.7391°W, 26.VII.2005, 11.VI.2007, R. P. Webster, mixed forest, u.v. light (4, RWC); same locality, habitat data, and collector but 23.IV.2008, collected during aerial flight between 15:00 to 18:00h (1, RWC); 15 km W of Tracy off Rt. 645, 45.6848°N, 66.8821°W, 8–15.VI.2009, 15–21.VI.2009, M.-A. Giguère, R. Webster, & V. Webster, old red pine forest, Lindgren funnel traps (4, AFC); 14 km WSW of Tracy, S of Rt. 645, 45.6741°N, 66.8661°W, 26.IV-10.V.2010, 10–26.V.2010, 30.VI-13.VII.2010, R. Webster C. MacKay & K. Burgess, old mixed forest with red and white spruce, red and white pine, balsam fir, eastern white cedar, red maple, and *Populus* sp., Lindgren funnel traps (5, AFC, RWC).

####### Collection and habitat data.

Adults were found in a mature hardwood forest with sugar maple (*Acer saccharum* Marsh.), American beech (*Fagus grandifolia* Ehrh.), white ash (*Fraxinus americana* L.), and butternut (*Juglans cinerea* L.), an old-growth northern hardwood forest with sugar maple and yellow birch (*Betula alleghaniensis* Britt.), an old silver maple (*Acer saccharinum* L.) swamp, an old-growth red pine forest, a mature red spruce (*Picea rubens* Sarg.) forest, an old eastern white cedar (*Thuja occidentalis* L.) swamp/forest, and old mixed forests. This species was captured in Lindgren funnel traps at all sites where these traps were used. Adults were also collected during an evening flight (between 15:00 and 18:00 h), and at an ultraviolet light. Adults were collected during April, May, June, July, and August.

####### Distribution in Canada and Alaska.

ON, QC, **NB** (Campbell 1991a). Casey (1898) used the number of antennal segments of the club to distinguish *Sphindus trinifer* (three-segmented club) from *Sphindus americanus* (two-segmented club) in his key to the American *Sphindus* species. However, Downie and Arnett (1996) and Majka (2010) used size and other characteristics such as color to separate *Sphindus americanus* (1.5 to 2.5 mm in length) from *Sphindus trinifer* (1.7 mm in length). These characteristics are variable in these two species and are, therefore, unreliable for use in distinguishing these species. The specimens reported above all possess a three-segmented club, a character of *Sphindus trinifer*. The adults from New Brunswick are, on average, larger (ranging from 1.7 to 2.0 mm in length) than the 1.7 mm given for the type specimen of *Sphindus trinifer* (from Toronto, Canada) in Casey’s original description. The specimens otherwise agree with the original description of *Sphindus trinifer*. Interestingly, *Sphindus americanus* was reported by Majka (2010) to be common and widespread in Nova Scotia. However, the specimen illustrated in his paper possesses a three-segmented club, a character of *Sphindus trinifer*. The Nova Scotia specimens should be re-examined to confirm their identity.

**Map 3.**
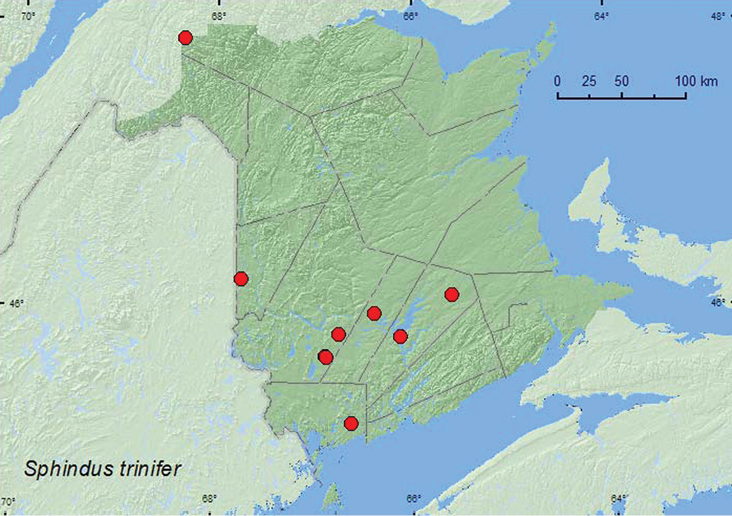
Collection localities in New Brunswick, Canada of *Sphindus trinifer*.

#### Family Erotylidae Latreille, 1802

The Erotylidae (and Endomychidae) of the Maritime provinces were reviewed by Majka (2007). *Triplax dissimulator* (Crotch) was reported from New Brunswick for the first time. Majka et al. (2010b) later reported *Acropteroxys gracilis* (Newman) (Languriinae Hope) from New Brunswick. The Erotylidae live in hard bracket fungi (Polyporacae) (Subfamilies Dacninae and Megalodacninae) and soft polypores and basidiomycetes (Tritominae) (Skelley et al. 1991; Skelley and McHugh 2002). Members of the Languriinae are stem borers on composites and legumes, and adults are usually collected on their host plants (Leschen and Skelley 2002b). Majka (2007) discussed the fungal associations of members of the Erotylidae from the Maritime provinces and the impact that forest management practices may have on the communities of forest fungi and the associated beetle species dependent on these fungi. Four species of Erotylidae were reported from New Brunswick by Majka (2007) and Majka et al. (2010b). Here, we add five species of Erotylidae to the Coleoptera faunal list of New Brunswick, including *Tritoma humeralis* Fabricius and *Tritoma sanguinipennis* (Say), which are new to the Maritime provinces (Table 1).

##### Subfamily Erotylinae Latreille, 1802

Tribe Dacnini Gistel, 1848

###### Dacne quadrimaculata

(Say, 1835)

http://species-id.net/wiki/Dacne_quadrimaculata

Map 4

####### Material examined.

**New Brunswick, Carleton Co.**, Jackson Falls, Bell Forest, 46.2200°N, 67.7231°W, 28.VI.2005, R. P. Webster, mature hardwood forest, u.v. light (1, RWC); same locality and habitat data but 12–19.VI.2008, R. P. Webster, Lindgren funnel traps (2, RWC); Meduxnekeag Valley Nature Preserve, 46.1907°N, 67.6740°W, 20.VI.2006, R. P. Webster, mixed forest, in partially dried *Pleurotus* species on dead standing trembling aspen (2, RWC); same locality but 46.1877°N, 67.6717°W, 2.IX.2008, R. P. Webster, hardwood forest, on slightly dried *Climacodon septentrionale* on sugar maple (4, RWC). **Sunbury Co.**, Burton near Sunpoke Lake, 45.7658°N, 66.5546°W, 20.VI.2007, R. P. Webster, red oak and red maple forest, on slightly dried *Pleurotus* sp. on dead standing poplar (1, RWC).

#######  Collection and habitat data.

In New Brunswick, adults of this species were collected in a mature hardwood forest with American beech, sugar maple, and ash, mixed forests, and an old red oak forest. Most individuals were collected from partially dried *Pleurotus* sp. on dead standing *Populus* sp. and on a slightly dried *Climacodon septentrionale* (Fr.) Kar. on a dead standing sugar maple. A few adults were also captured in Lindgren funnel traps and at an ultraviolet light. Skelley et al. (1991) reported that larvae of this species feed in a variety of hard and soft basidiomycete bracket fungi, including *Pleurotus* sp. In New Brunswick, adults were collected during June and September.

####### Distribution in Canada and Alaska.

MB, ON, QC, **NB**, NS (Campbell 1991b; Majka 2007).

**Map 4.**
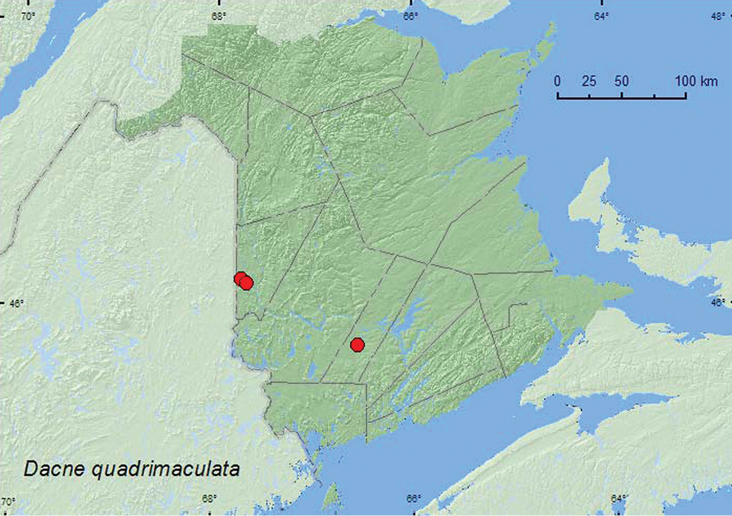
Collection localities in New Brunswick, Canada of *Dacne quadrimaculata*

##### Tribe Tritomini Curtis, 1834

###### Triplax macra

LeConte 1854

http://species-id.net/wiki/Triplax_macra

Map 5

####### Material examined.

**New Brunswick, Carleton Co.**, Jackson Falls, Bell Forest, 46.2200°N, 67.7231°W, 28.VII.2008, 18.VIII.2008, 20.IX.2008, mature hardwood forest, in *Hapalophilus nitulans* (a fleshy polypore fungus) (18, NBM, RWC); same locality and forest type but 12–19.VI.2008, 12–19.VII.2008, R. P. Webster, Lindgren funnel traps (2, AFC); same locality and habitat data but 21–28.VI.2009, Webster & M.-A. Giguère, Lindgren funnel traps (2, AFC). **Queens Co.**, Cranberry Lake P.N.A., 46.1125°N, 65.6075°W, 13–20.VII.2011, M. Roy & V. Webster, old red oak forest, Lindgren funnel trap (1, NBM). **Restigouche, Co.**, Dionne Brook P.N.A., 47.9030°N, 68.3503°W, 30.V-15.VI.2011, M. Roy & V. Webster, old-growth northern hardwood forest, Lindgren funnel traps (4, AFC, NBM); same locality and collectors but 47.9064°N, 68.3441°W, 31.V-15.VI.2011, 27.VI–14.VII.2011, old-growth northern hardwood forest, Lindgren funnel traps (2, NBM, RWC). **York Co.**, 15 km W of Tracy off Rt. 645, 45.6848°N, 66.8821°W, 10–30.VIII.2010, R. Webster & K. Burgess, old red pine forest, Lindgren funnel trap (1, AFC)

####### Collection and habitat data.

A long series of adults of *Triplax macra* were collected from *Hapalophilus nitulans* (Fr.) Kar. (a fleshy polypore fungus) in a mature hardwood forest. Additional adults were captured in Lindgren funnel traps at this same site and from funnel traps deployed in an old red pine forest, an old red oak forest, an old-growth northern hardwood forest, and an old-growth white spruce (*Picea glauca* (Moench) Voss) and balsam fir (*Abies balsamea* (L.) Mill.) forest. Adults were captured during July, August, and September. Skelley et al. (1991) reported this species from two *Inonotus* sp. and *Pleurotus ostreatus* Fr.

####### Distribution in Canada and Alaska.

MB, ON, QC, **NB**, NS (Campbell 1991b; Majka 2007).

**Map 5.**
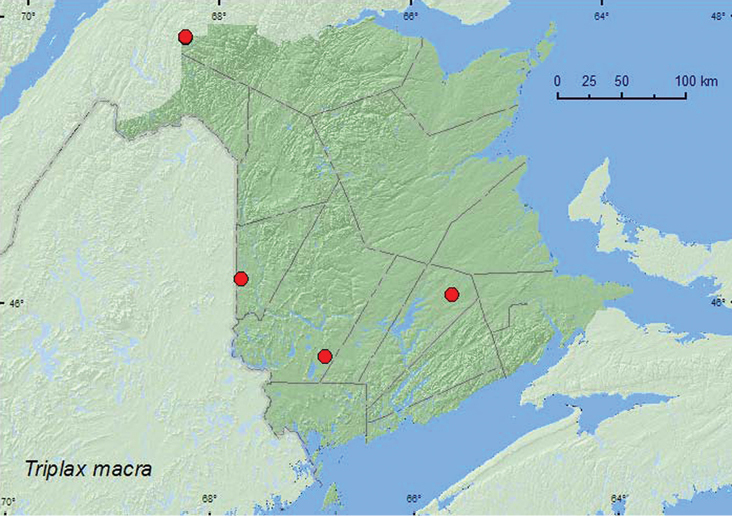
Collection localities in New Brunswick, Canada of *Triplax macra*.

###### Tritoma humeralis

Fabricius, 1801**

http://species-id.net/wiki/Tritoma_humeralis

Map 6

####### Material examined.

**New Brunswick, Sunbury Co.**, Acadia Research Forest, 46.0173°N, 66.3741°W, 18.VI.2007, R. P. Webster, 8.5-year-old regenerating mixed forest, in gilled mushroom on stump (sun-exposed) (1, RWC).

####### Collection and habitat data.

One adultof this species was collected during June in a gilled mushroom on a sun-exposed stump in an 8.5-year-old regenerating mixed forest.

####### Distribution in Canada and Alaska.

ON, QC, **NB** (Campbell 1991b).

**Map 6.**
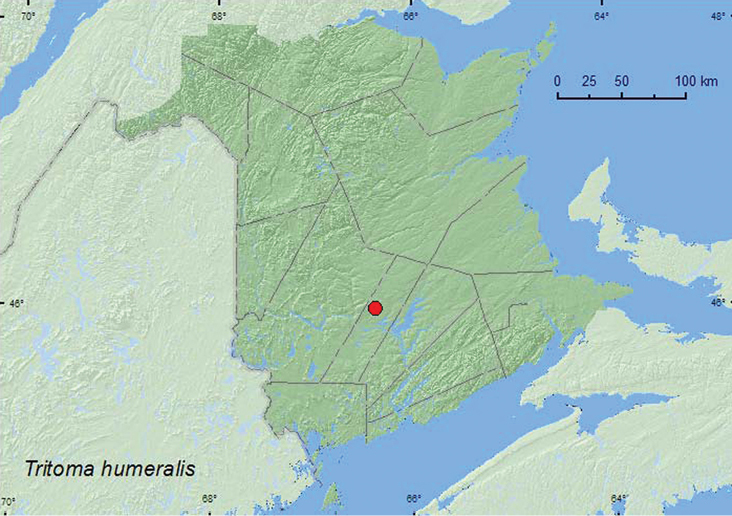
Collection localities in New Brunswick, Canada of *Tritoma humeralis*.

###### Tritoma pulchra

Say, 1826

http://species-id.net/wiki/Tritoma_pulchra

Map 7

####### Material examined.

**New Brunswick, Carleton Co.**, Jackson Falls, “Bell Forest”, 46.2210°N, 67.7210°W, 12.VII.2004, K. Bredin, J. Edsall, & R. Webster, mature mixed forest, sweeping foliage (1, RWC); same locality but 46.2200°N, 67.7231°W, 27.VI–5.VII.2008, R. P. Webster, mature hardwood forest, Lindgren funnel trap (1, AFC); same locality and habitat data but 1–8.VI.2009, 8–16.VI.2009, 21–28.VI.2009, 19–31.VII.2009, 31.VII–7.VIII.2009, 7–12.VIII.2009, Webster & M.-A. Giguère, Lindgren funnel traps (6, AFC); Meduxnekeag Valley Nature Preserve, 46.1907°N, 67.6740°W, 8.VIII.2006, R. P. Webster, mixed forest, in slightly decayed polypore fungus on log (5, RWC); Hartland, Becaguimec Island (in Saint John River), 46.3106°N, 67.5372°W, 16.IX.2006, R. P. Webster, hardwood forest, in fleshy polypore fungi on dead standing *Populus* sp. (4, NBM, RWC). **Charlotte Co.**, 10 km NW of New River Beach, 45.2110°N, 66.6170°W, 26.VII-10.VIII.2010, R. Webster & C. MacKay, old growth eastern white cedar forest, Lindgren funnel trap (1, AFC). **Northumberland Co.**, Goodfellow Brook P.N.A. , 46.8943°N, 65.3796°W, 23.V.2007, R. P. Webster, old growth eastern white cedar swamp, in litter with grasses and moss on hummock near water (1, RWC). **Queens Co.**, Cranberry Lake P.N.A., 46.1125°N, 65.6075°W, 18–25.VI.2009, 25.VI–1.VII.2009, 21–28.VII.2009, 28.VII-6.VIII.2009, R. Webster & M.-A. Giguère, mature red oak forest, Lindgren funnel traps (5, AFC). **Restigouche, Co.**, Dionne Brook P.N.A., 47.9064°N, 68.3441°W, 15–27.VI.2011, M. Roy & V. Webster, old-growth white spruce and balsam fir forest, Lindgren funnel trap (1, NBM). **Sunbury Co.**, Acadia Research Forest, 45.9866°N, 66.3841°W, 13–21.VII.2009, 21–29.VII.2009, 29.VII-4.VIII.2009, R. Webster & M.-A. Giguère, mature (110 year-old) red spruce forest with scattered red maple and balsam fir, Lindgren funnel trap (5, AFC). **York Co.**, Charters Settlement, 45.8286°N, 66.7365°W, 13–17.VII.2008, R. P. Webster, mature mixed forest, Lindgren funnel trap (1, NBM); Rt. 645 at Beaver Brook, 45.6860°N, 66.8668°W, 13.VIII.2008, R. P. Webster, sedge marsh, on flowers of *Spiraea alba* (1, NBM); 15 km W of Tracy off Rt. 645, 45.6848°N, 66.8821°W, 7–14.VII.2009, R. Webster & M.-A. Giguère, old red pine forest, Lindgren funnel trap (1, AFC); 14 km WSW of Tracy, S of Rt. 645, 45.6741°N, 66.8661°W, 26.V–2.VI.2010, 16–30.VI.2010, R. Webster & C. MacKay, old mixed forest with red and white spruce, red and white pine, balsam fir, eastern white cedar, red maple, and *Populus* sp., Lindgren funnel traps (2, AFC).

####### Collection and habitat data.

In New Brunswick, *Tritoma pulchra* was found in a variety of forest types, such as mature hardwood forests, an old red oak forest, mixed forests, an old red spruce forest, an old red pine forest, an old-growth white spruce and balsam fir forest, and old-growth eastern white cedar forests. Most adults were collected from soft polypore fungi on logs and dead standing trees or captured in Lindgren funnel traps. A few individuals were collected by sweeping vegetation or sifting litter. One adult was found on flowers of meadow sweet (*Spiraea alba* Du Roi) in a sedge marsh. Adults were collected during June, July, August, and September.

####### Distribution in Canada and Alaska.

ON, QC, **NB**, NS (Campbell 1991b).

**Map 7.**
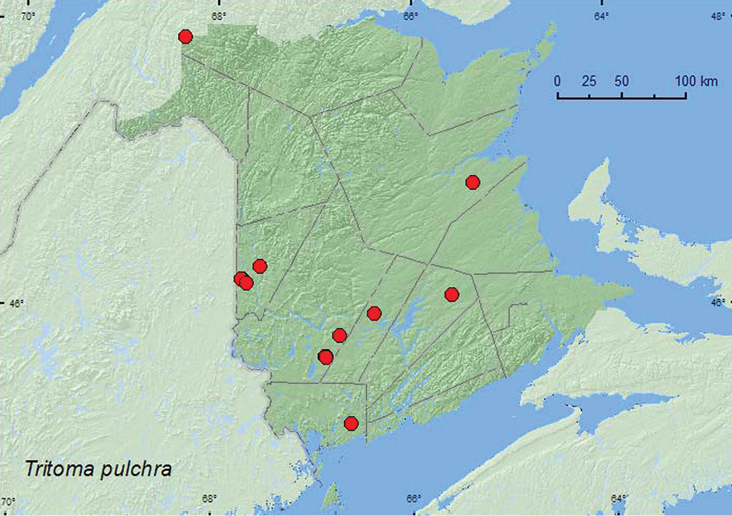
Collection localities in New Brunswick, Canada of *Tritoma pulchra*.

###### Tritoma sanguinipennis

(Say, 1825)**

http://species-id.net/wiki/Tritoma_sanguinipennis

Map 8

####### Material examined.

**New Brunswick, Queens Co.**, Cranberry Lake P.N.A., 46.1125°N, 65.6075°W, 2.IX.2009, R. P. Webster, mature red oak forest, fleshy polypore fungus on side of log (1, RWC).

####### Collection and habitat data.

The only specimen known from New Brunswickwas collected in a soft polypore fungus on the side of a log in September.

####### Distribution in Canada and Alaska.

ON, QC, **NB** (Campbell 1991b).

**Map 8.**
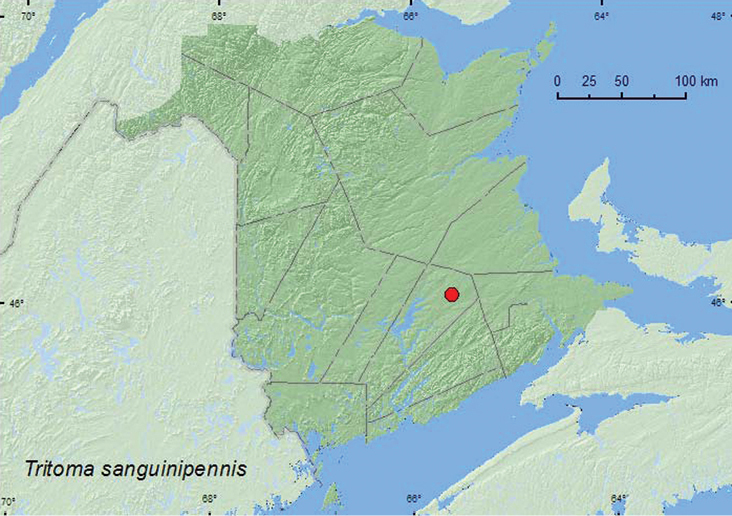
Collection localities in New Brunswick, Canada of *Tritoma sanguinipennis*.

#### Family Monotomidae Laporte, 1840

Most members of the family Monotomidae (the root-eating beetles) are subcortical and are considered predators of xylophagous insects, such as scolytine larvae, although some may feed on fungi and their by-products (Bousquet 2002). Some species (*Monotoma* species) live in decaying vegetable matter and often are found in compost heaps (Bousquet and Laplante 2000; Bousquet 2002). The Monotomidae of the Maritime provinces were recently reviewed by Majka and Bousquet (2010). Six species were reported from New Brunswick. Here, we add three additional species to the faunal list, including *Pycnotomina cavicollis* (Horn), which is newly recorded for the Maritime provinces (Table 1).

##### Subfamily Rhizophaginae Redtenbacher, 1845

###### Rhizophagus dimidiatus

Mannerheim, 1843

http://species-id.net/wiki/Rhizophagus_dimidiatus

Map 9

####### Material examined.

**Additional New Brunswick records. Carleton Co.**, Jackson Falls, Bell Forest, 46.2200°N, 67.7231°W, 6.V.2007, 7.VI.2007, R. P. Webster, mature hardwood forest, on fleshy polypore (bracket) fungi on dead standing beech (2, RWC); same locality but 4–12.VI.2008, 12–19.VI.2008, 27.VI-5.VII.2008, R. P. Webster, mature hardwood forest, Lindgren funnel traps (8, AFC, RWC); same locality and habitat data but 20–26.V.2009, 1–8.VI.2009, 16–21.VI.2009, 21–28.VI.2009, Webster & M.-A. Giguère, Lindgren funnel traps (4, AFC, RWC). **Queens Co.**, Cranberry Lake P.N.A, 46.1125°N, 65.6075°W, 5–11.VI.2009, 11–18.VI.2009, 18–25.VI.2009, 25.VI–1.VII.2009, R. Webster & M.-A. Giguère, old red oak forest, Lindgren funnel traps (11, AFC). **Restigouche Co.**, Dionne Brook P.N.A., 47.9030°N, 68.3503°W, 30.V–15.VI.2011, M. Roy & V. Webster, old-growth northern hardwood forest, Lindgren funnel traps (2, AFC, NBM); same locality and collectors but 47.9064°N, 68.3441°W, 31.V–15.VI.2011, 27.VI–14.VII.2011, old-growth white spruce and balsam fir forest, Lindgren funnel traps (2, AFC, NBM). **Sunbury Co.**, Acadia Research Forest, 45.9866°N, 66.3841°W, 2–9.VI.2009, 24–30.VI.2009, R. Webster & M.-A. Giguère, mature (110-year-old) red spruce forest with scattered red maple and balsam fir, Lindgren funnel traps (2, AFC). **York Co.**, Charters Settlement, 45.8286°N, 66.7365°W, 6.VI.2007, R. P. Webster, mature red spruce and red maple forest, under scolytid infested bark of red spruce (2, RWC); 15 km W of Tracy off Rt. 645, 45.6848°N, 66.8821°W, 8–15.VI.2009, 15–21.VI.2009, 20–29.VII.2009, R. Webster & M.-A. Giguère, old red pine forest, Lindgren funnel traps (4, AFC); 14 km WSW of Tracy, S of Rt. 645, 45.6741°N, 66.8661°W, 10–26.V.2010, R. Webster & C. MacKay, old mixed forest with red and white spruce, red and white pine, balsam fir, eastern white cedar, red maple, and *Populus* sp., Lindgren funnel trap (1, AFC).

####### Collection and habitat data.

Most adults from New Brunswick were captured in Lindgren funnel traps. This species occurred in various forest types, including mature hardwood forests, an old-growth northern hardwood forest, an old red oak forest, old mixed forests, an old red pine forest, and an old-growth white spruce and balsam fir forest. Specimens with specific habitat data were collected from under scolytine-infested bark of red spruce and on fleshy polypore (bracket) fungi on dead standing American beech trees. Bousquet (1990) reported this species from under bark of deciduous (*Acer* sp., *Betula* sp., *Fagus* sp.) and coniferous (*Pinus* sp., *Larix* sp., *Picea* sp.) trees in eastern North America. Adults were collected during May, June, and July in New Brunswick.

####### Distribution in Canada and Alaska.

AK, YK, BC, AB, ON, QC, NB, NS, NF (Bousquet 1990; Majka and Bousquet 2010). *Rhizophagus dimidiatus* was first reported from New Brunswick by Majka and Bousquet (2010) on the basis of one specimen from Chatham, Northumberland Co., collected by P. Kaanar (in CNC). This species is widespread and common in New Brunswick.

**Map 9.**
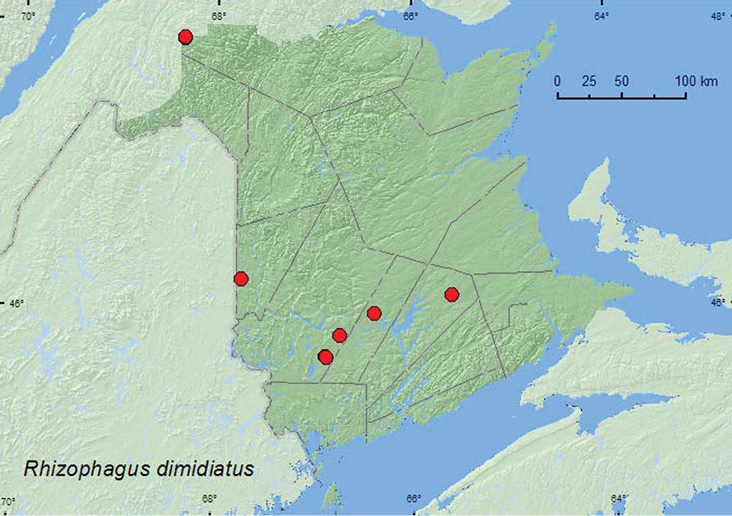
Collection localities in New Brunswick, Canada of *Rhizophagus dimidiatus*.

###### Rhizophagus minutus rotundicollis

Bousquet, 1990

http://species-id.net/wiki/Rhizophagus_minutus_rotundicollis

Map 10

####### Material examined.

**New Brunswick, York Co.**, Charters Settlement, 45.8395°N, 66.7391°W, 20.IV.2004, R. P. Webster, mixed forest, compost, decaying vegetables (1, RWC); same locality data but 23.IV.2008, 4.IV.2010, R. P. Webster, mixed forest opening, in flight between 15:00 and 18:00 h (2, RWC); Charters Settlement, 45.8340°N, 66.7450°W, 29.III.2006, R. P. Webster, mixed forest, margin of vernal pond in leaf litter (1, RWC).

####### Collection and habitat data.

Bousquet (1990) reported this subspecies from balsam fir and white spruce. Specimens from New Brunswick were collected from decaying vegetables, in leaf litter on the margin of a vernal pond, and in flight between 15:00 and 18:00 h in a mixed forest opening. Adults were captured during March and April.

####### Distribution in Canada and Alaska.

ON, QC, **NB**, NS, NF (Bousquet 1990; Majka and Bousquet 2010).

**Map 10.**
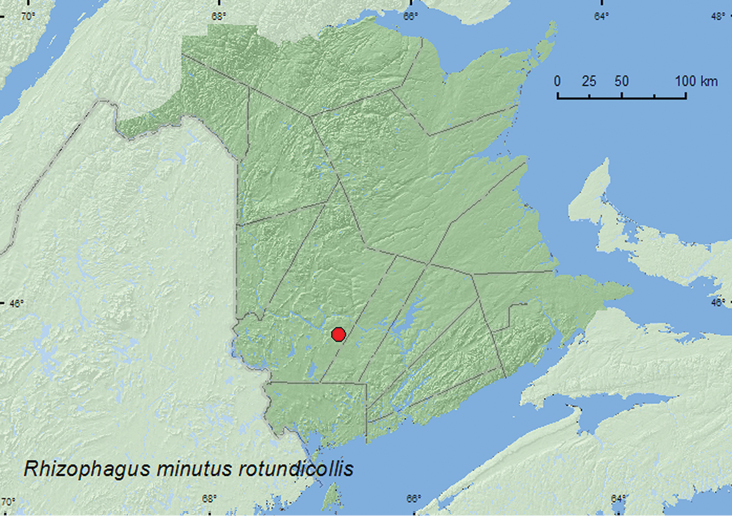
Collection localities in New Brunswick, Canada of *Rhizophagus minutus rotundicollis*.

###### Rhizophagus remotus

LeConte, 1866

http://species-id.net/wiki/Rhizophagus_remotus

Map 11

####### Material examined.

**New Brunswick, Carleton Co.**, Richmond, near Hovey Hill P.N.A., 46.1155°N, 67.7631°W 24.V.2005, R. P. Webster, clear-cut (hardwood forest), under bark of *Populus* sp. (6, NBM, RWC); Jackson Falls, Bell Forest, 46.2200°N, 67.7231°W, 23–28.IV.2009, R. Webster & M.-A. Giguère, mature hardwood forest, Lindgren funnel traps (2, AFC). **Queens Co.**, Cranberry Lake P.N.A, 46.1125°N, 65.6075°W, 5–11.VI.2009, 25.VI-1.VII.2009, R. Webster & M.-A. Giguère, old red oak forest, Lindgren funnel traps (3, AFC, RWC). **York Co.**, Charters Settlement, 45.8331°N, 66.7410°W, 29.V.2007, R. P. Webster, mature red spruce forest, under bark of *Populus* sp. (7, NBM, RWC); same locality, forest type and collector, 1.IV.2007, under bark of stump sticking out of snow (1, NBM); Charters Settlement, 45.8395°N, 66.7391°W, 23.IV.2008, R. P. Webster, mixed forest opening, in flight between 15:00 and 18:00 h (1, RWC); 15 km W of Tracy off Rt. 645, 45.6848°N, 66.8821°W, 1–8.VI.2009, 15–21.VI.2009, 14–20.VII.2009, R. Webster & M.-A. Giguère, old red pine forest, Lindgren funnel traps (3, AFC); 14 km WSW of Tracy, S of Rt. 645, 45.6741°N, 66.8661°W, 26.IV–10.V.2010, 26.V–2.VI.2010, R. Webster & C. MacKay, old mixed forest with red and white spruce, red and white pine, balsam fir, eastern white cedar, red maple, and *Populus* sp., Lindgren funnel traps (2, AFC).

####### Collection and habitat data.

This species has been reported under bark of pine and various *Populus* species, but most commonly from under bark of *Populus tremuloides* Michx. (Bousquet 1990). Adults in New Brunswick were taken from under bark of *Populus tremuloides* and under bark of a *Populus* stump sticking out of snow in early April, and were collected with an aerial net during an evening flight. Other individuals were captured in Lindgren funnel traps deployed in a mature hardwood forest, an old red oak forest, an old red pine forest, and in an old mixed forest. Adults were captured during April, May, June, and July.

####### Distribution in Canada and Alaska.

AK, BC, AB, MB, ON, QC, **NB**, NS (Bousquet 1990).

**Map 11.**
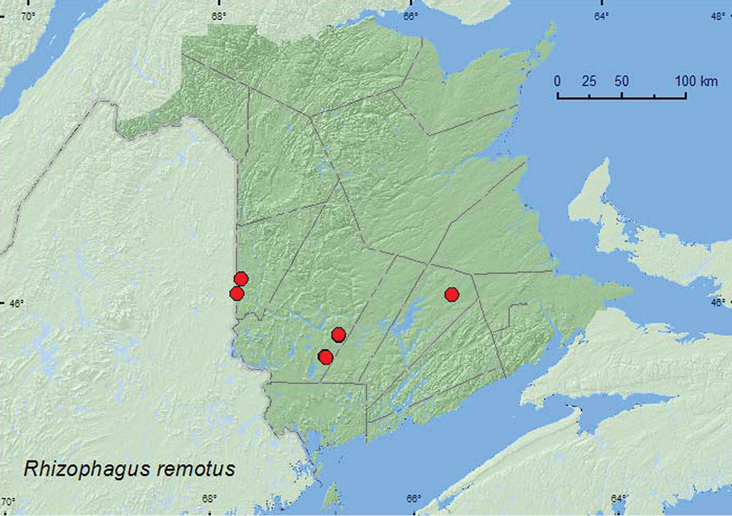
Collection localities in New Brunswick, Canada of *Rhizophagus remotus*.

##### Subfamily Monotominae Laporte, 1840

###### Pycnotomina cavicollis

(Horn, 1879)**

http://species-id.net/wiki/Pycnotomina_cavicollis

Map 12

####### Material examined.

**New Brunswick, Carleton Co.**, Jackson Falls, Bell Forest, 46.2200°N, 67.7231°W, 4–12.VI.2008, 12–19.VI.2008, R. P. Webster, mature hardwood forest, Lindgren funnel traps (12, AFC, RWC).

####### Collection and habitat data.

All adults of this species from New Brunswick were captured in Lindgren funnel traps deployed in a mature hardwood forest with sugar maple, white ash, butternut, American beech, and scattered eastern hemlock (*Tsuga canadensis* (L.) Carr.). Adults were captured during June.

####### Distribution in Canada and Alaska.

ON, QC, **NB** (Bousquet 1991a).

**Map 12.**
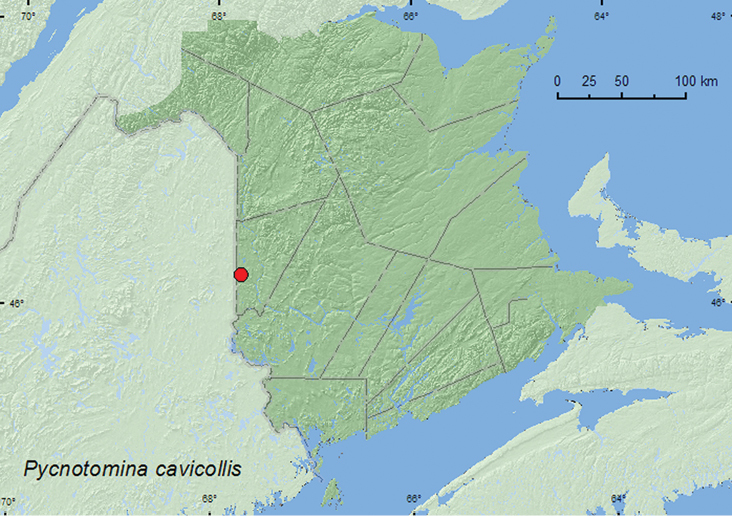
Collection localities in New Brunswick, Canada of *Pycnotomina cavicolle*.

#### Family Cryptophagidae Kirby, 1826

The Cryptophagidae (silken fungus beetles) usually occur in moist decaying habitats that promote fungal growth, such as leaf litter and rotting wood, where they feed on fungal hyphae, spores, and conidia (Leschen and Skelley 2002a). Some species are saprophagous, while others can be found on flowers. *Antherophagus* species are phoretic on *Bombus* bees and are found in the nests or at flowers (Bousquet 1989, Leschen and Skelley 2002a). The Cryptophagidae of Atlantic Canada were reviewed by Majka et al. (2010a) (Atomariinae) and Majka and Langor (2010) (Cryptophaginae). Seven species of *Atomaria* (Atomariinae) (Majka et al. 2010a) and five species of Cryptophaginae (Majka and Langor 2010) were reported from New Brunswick. However, the record of *Antherophagus convexulus* LeConte reported in Bousquet (1991b) was considered provisional by Majka and Langor (2010) due to lack of a supporting voucher specimen. Below, we report six additional species of Cryptophaginae from New Brunswick and confirm the presence of *Antherophagus convexulus* in the province (Table 1).*Cryptophagus pilosus* Gyllenhal and *Myrmedophila americana* (LeConte) are new to the Maritime provinces.

##### Subfamily Cryptophaginae Kirby, 1826. Tribe Cryptophagini Kirby, 1826

###### Antherophagus convexulus

LeConte, 1863

http://species-id.net/wiki/Antherophagus_convexulus

Map 13

####### Material examined.

**Additional New Brunswick records. Queens Co.**, Cranberry Lake P.N.A., 46.1125°N, 65.6075°W, 6.VIII.2009, M.-A. Giguère, mature red oak forest, on flowers of *Spiraea alba* (1, RWC).

####### Collection and habitat data.

Adults of *Antherophagus* sp. are phoretic on *Bombus* spp. and are often found in their nests or on flowers (Bousquet 1989; Leschen and Skelley 2002a). The specimen of *Antherophagus convexulus* from New Brunswick was found on flowers of *Spiraea alba* DuRoi during early August.

####### Distribution in Canada and Alaska.

ON, QC, NB, NS (Bousquet 1991b). Majka and Langor (2010) were unable to locate any voucher specimens or published records to support the record for New Brunswick in Bousquet (1991b), but provisionally retained this species for the province. The record above confirms the presence of this species for New Brunswick.

**Map 13.**
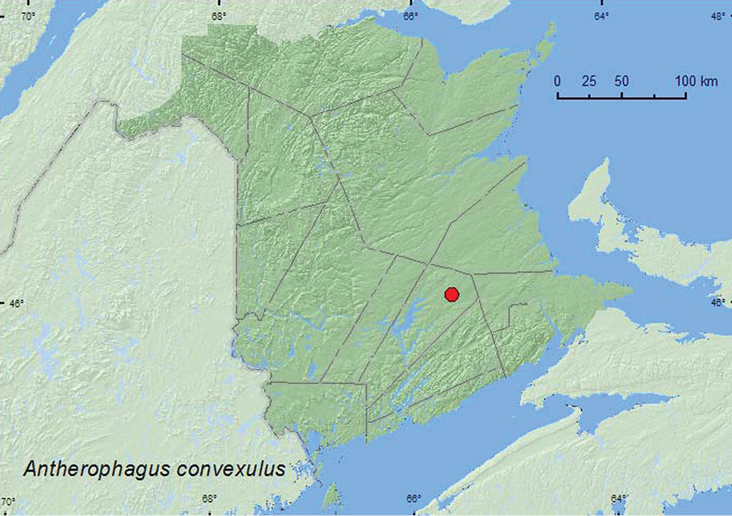
Collection localities in New Brunswick, Canada of *Antherophagus convexulus*

###### Cryptophagus acutangulus

Gyllenhal, 1827

http://species-id.net/wiki/Cryptophagus_acutangulus

Map 14

####### Material examined.

**New Brunswick, York Co.**, Charters Settlement, 45.8395°N, 66.7391°W, 5.V.2006, R. P. Webster, mixed forest, compost (decaying vegetable matter) (1, RWC); same locality, collector and forest type, 4.IV.2010, collected with aerial net during evening flight between 16:30 h and 19:00 h (1, RWC); 14 km WSW of Tracy, S of Rt. 645, 45.6741°N, 66.8661°W, 25.IV–10.V.2010, R. Webster & C. MacKay, old mixed forest with red and white spruce, red and white pine, balsam fir, eastern white cedar, red maple, and *Populus* sp., Lindgren funnel trap (1, AFC).

####### Collection and habitat data.

In North America, the Holarctic*Cryptophagus acutangulus* has been reported from *Solidago*, on lumber, on *Salix*, on *Pinus ponderosa*, in stored grain, from grain elevators, at light, and collected during evening flight (based on label data) (Woodroffe and Coombs 1961). New Brunswick specimens were collected from compost, during evening flight, and from a Lindgren funnel trap deployed in an old mixed forest. Adults were captured during April and May.

####### Distribution in Canada and Alaska.

AK, BC, AB, MB, ON, QC, **NB**, NS, NF (Bousquet 1991b; Majka and Langor 2010).

**Map 14.**
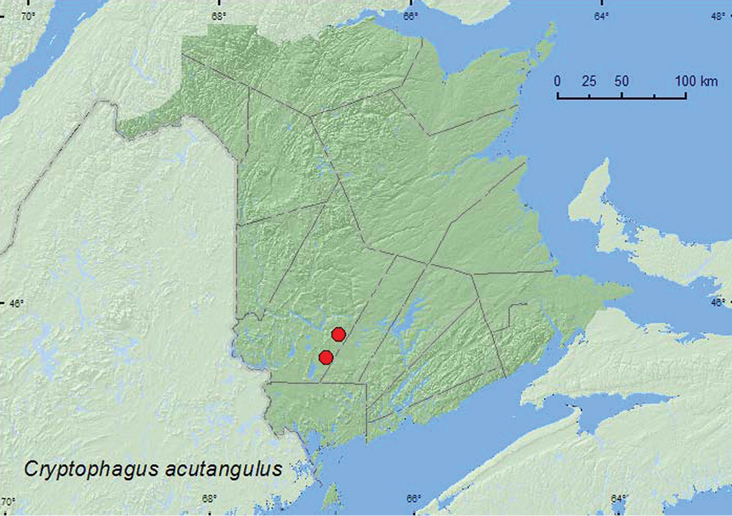
Collection localities in New Brunswick, Canada of *Cryptophagus acutangulus*.

###### Cryptophagus pilosus

Gyllenhal, 1827**

http://species-id.net/wiki/Cryptophagus_pilosus

Map 15

####### Material examined.

**New Brunswick, York Co.**, Fredericton, 7.I.1922, R. P. Gorham, stored turnips (1, AFC).

####### Collection and habitat data.

The single adult from New Brunswick was collected from stored turnips in January. Woodroffe and Coombs (1961) reported this Holarctic species from stored products and vegetable refuse.

####### Distribution in Canada and Alaska.

BC, MB, ON, **NB** (Bousquet 1991b).

**Map 15.**
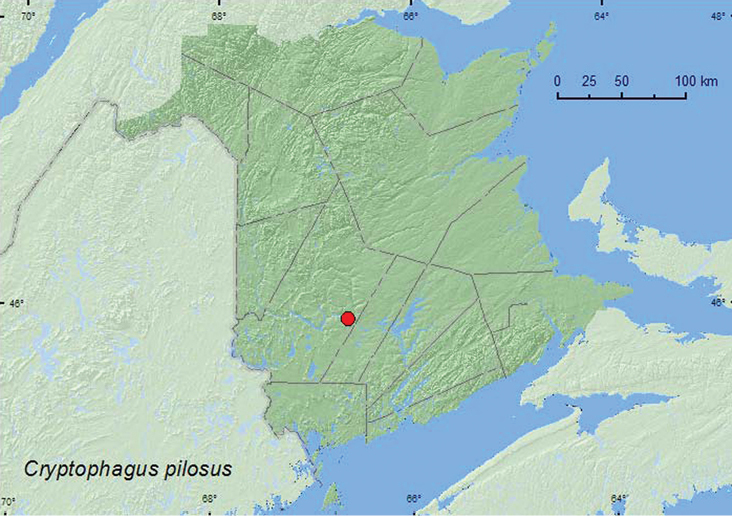
Collection localities in New Brunswick, Canada of *Cryptophagus pilosus*.

###### Cryptophagus mainensis

Casey, 1924

http://species-id.net/wiki/Cryptophagus_mainensis

Map 16

####### Material examined.

**New Brunswick, Carleton Co.**, Jackson Falls, Bell Forest, 46.2200°N, 67.7231°W, 25.VIII-2.IX.2008, R. P. Webster, mature hardwood forest, Lindgren funnel trap (1, RWC). **Queens Co.**, Cranberry Lake P.N.A., 46.1125°N, 65.6075°W, 1–10.VII.2009, R. Webster & M.-A. Giguère, mature red oak forest, Lindgren funnel trap (1, RWC).

####### Collection and habitat data.

*Cryptophagus mainensis* was reported from red spruce and hemlock forests in Nova Scotia (Majka and Langor 2010). The specimens from New Brunswick were captured in Lindgren funnel traps deployed in a mature hardwood forest with American beech, sugar maple, and white ash, and an old red oak forest. Adults were collected during July, August, and September.

####### Distribution in Canada and Alaska.

**NB**, NS, NF (Majka and Langor 2010). Majka and Langor (2010) reported this species for the first time for Canada from Nova Scotia and Newfoundland.

**Map16.**
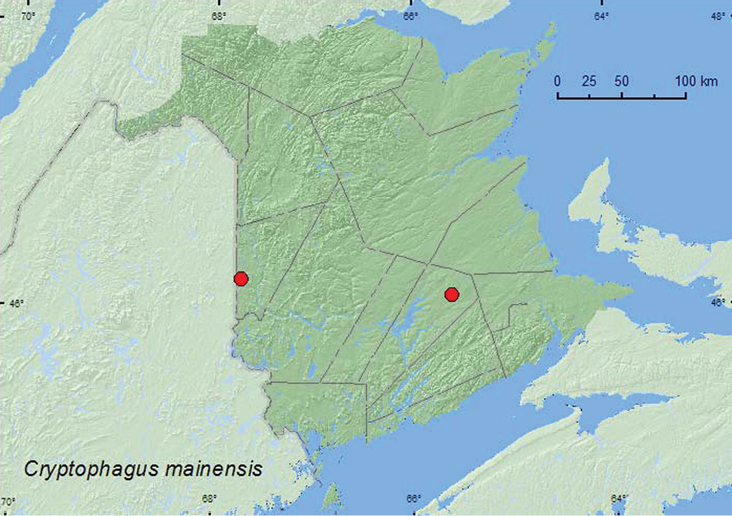
Collection localities in New Brunswick, Canada of *Cryptophagus mainensis*.

###### Henoticus serratus

(Gyllenhal, 1808)

http://species-id.net/wiki/Henoticus_serratus

Map 17

####### Material examined.

**New Brunswick, Queens Co.**, Cranberry Lake P.N.A., 46.1125°N, 65.6075°W, 24.IV-5.V.2009, 5–12.V.2009, 12–21.V.2009, 21–27.V.2009, 27.V–5.VI.2009, 5–11.VI.2009, 11–18.VI.2009, 18–25.VI.2009, R. Webster & M.-A. Giguère, mature red oak forest, Lindgren funnel traps (17, AFC, NBM, RWC). **York Co.**, McAdam, Georgia Pacific Plywood Mill, 19.V.1978, F.A.T. and U.P.N., on radiata pine, F.I.D.S., 78–2-2051–13 (1, AFC); Charters Settlement, 45.8395°N, 66.7391°W, 5.IX.2006, R. P. Webster, mixed forest, among moldy corncobs and cornhusks (1, RWC).

####### Collection and habitat data.

Adults of *Henoticus* occur in leaf litter, fungi, under bark, on leaves of trees and shrubs (Bousquet 1989). Majka and Langor (2010) noted that *Henoticus serratus* were collected from natural habitats in Nova Scotia, such as red spruce and red oak forests. Most specimens from New Brunswick were collected from Lindgren funnel traps deployed in a mature red oak forest. One individual was collected from among moldy corncobs and cornhusks near a mixed forest. Adults were collected during April, May, June, and September.

####### Distribution in Canada and Alaska.

AK, BC, MB, ON, QC, **NB**, NS, NF (Bousquet 1991b; Majka and Langor 2010).

**Map 17.**
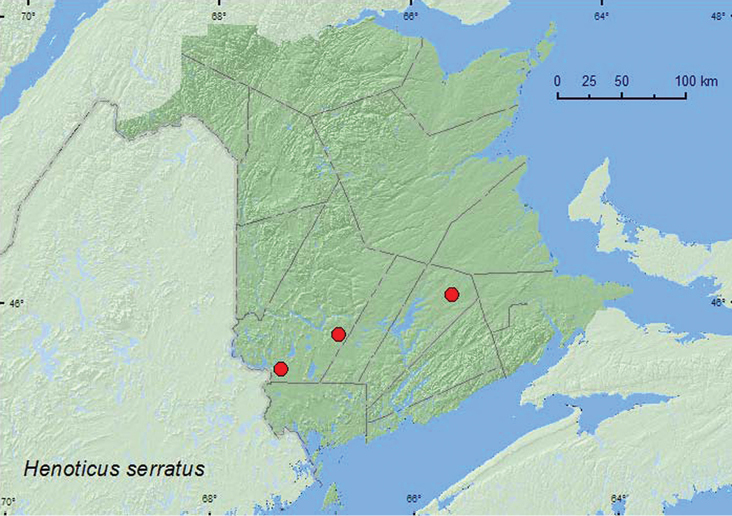
Collection localities in New Brunswick, Canada of *Henoticus serratus*.

###### Henotiderus centromaculatus

Reitter, 1877

http://species-id.net/wiki/Henotiderus_centromaculatus

Map 18

####### Material examined.

**New Brunswick, Carleton Co.**, Jackson Falls, Bell Forest, 46.2200°N, 67.7231°W, 6.V.2007, R. P. Webster, mature hardwood forest, under bark of fungus covered beech log (9, NBM, RWC); same locality, collector, and habitat data but 4–12.VI.2008, Lindgren funnel trap (1, AFC); same locality data and habitat but 22–28.IV.2009, 28.IV-9.V.2009, 1–8.VI.2009, Webster & M.-A. Giguère, Lindgren funnel traps (5, AFC); near Belleville, 1.3 km E jct. Rt. 640 & Plymouth Rd., 46.1867°N, 67.6817°W, 7.V.2008, R. P. Webster, old hardwood forest, in fleshy (shelf) polypore fungi on beech log (1 (many individuals observed), NBM). **Charlotte Co.**, 10 km NW of New River Beach, 45.2110°N, 66.6170°W, 31.V–15.VI.2010, R. Webster & C. MacKay, old growth eastern white cedar forest, Lindgren funnel trap (1, AFC). **Gloucester Co.**, near Black Rock, 47.7395°N, 65.2545°W, 8.VI.2006, R. P. Webster, eastern white cedar swamp, near slime mold under bark (of *Populus* log) (1, RWC). **Queens Co.**, Cranberry Lake P.N.A., 46.1125°N, 65.6075°W, 24.IV–5.V.2009, 5–12.V.2009, R. Webster & M.-A. Giguère, mature red oak forest, Lindgren funnel traps (6, AFC). **Restigouche Co.**, NE of jct. Little Tobique River and Red Brook, 47.4502°N, 67.0578°W, 24.V.2007, R. P. Webster, old-growth eastern white cedar swamp, under bark of *Populus* log (1, RWC); Dionne Brook P.N.A., 47.9064°N, 68.3441°W, 31.V–15.VI.2011, M. Roy & V. Webster, old-growth white spruce and balsam fir forest, Lindgren funnel trap (1, NBM). **Sunbury Co.**, Acadia Research Forest, 45.9866°N, 66.3841°W, 8–13.V.2009, 13–18.V.2009, 8–13.VII.2009, R. Webster & M.-A. Giguère, mature (110-year-old) red spruce forest with scattered red maple and balsam fir, Lindgren funnel traps (9, AFC). **York Co.**, Charters Settlement, 45.8286°N, 66.7365°W, 3.VI.2007, R. P. Webster, mature red spruce forest, under bark of red spruce (1, RWC); 15 km W of Tracy off Rt. 645, 45.6848°N, 66.8821°W, 22–25.IV.2009, 4–11.V.2009, 11–19.V.2009, 19–25.V.2009, 25.V–1.VI.2009, 15–21.VI.2009, R. Webster & M.-A. Giguère, old red pine forest, Lindgren funnel traps (6, AFC); 14 km WSW of Tracy, S of Rt. 645, 45.6741°N, 66.8661°W, 25.IV–10.V.2010, R. Webster & C. MacKay, old mixed forest with red and white spruce, red and white pine, balsam fir, eastern white cedar, red maple, and *Populus* sp., Lindgren funnel trap (1, AFC).

####### Collection and habitat data.

Bousquet (1989) reported that *Henotiderus* occur in leaf litter, and various fungi such as *Polyporus*, *Pleurotus* and *Fomes* in forests. Most specimens of *Henotiderus centromaculatus*from Nova Scotia were found in red spruce forests (Majka and Langor 2010). In New Brunswick, this species was found in various of forest types including mature hardwood forests, an old red oak forest, an old mixed forest, mature red spruce forests, an old red (180-year-old) pine forest, an old-growth white spruce and balsam fir forest (boreal forest), and eastern white cedar forests. Adults were found under bark of a fungus-covered beech log, under bark of a *Populus* log, under bark of a red spruce, near slime mold under bark of *Populus* sp., and in fleshy (shelf) polypore fungi on an American beech log. This species was frequently captured in Lindgren funnel traps. Adults were captured during April, May, June, and July.

####### Distribution in Canada and Alaska.

AK, NT, AB, ON, QC, **NB**, NS (Bousquet 1991b; as *Henotiderus obesulus* (Casey)).

**Map 18.**
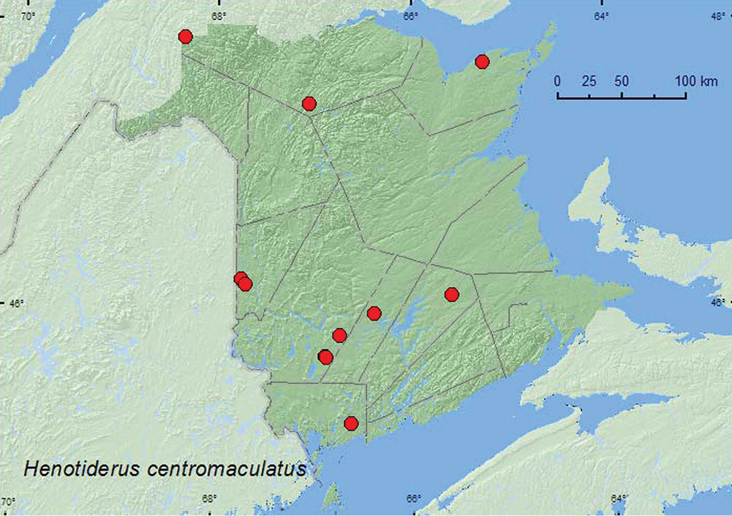
Collection localities in New Brunswick, Canada of *Henotiderus centromaculatus*.

###### Myrmedophila americana

(LeConte, 1879)**

http://species-id.net/wiki/Myrmedophila_americana

Map 19

####### Material examined.

**New Brunswick, Restigouche Co.**, Dionne Brook P.N.A., 47.9064°N, 68.3441°W, 30.V–15.VI.2011, M. Roy & V. Webster, old-growth white spruce and balsam fir forest, Lindgren funnel trap (1, NBM); same locality and collectors but 47.9030°N, 68.3503°W, 27.VI–14.VII.2011, old-growth northern hardwood forest, Lindgren funnel trap (1, RWC).

####### Collection and habitat data.

This species is myrmecophilous and associated with *Formica* sp. (Bousquet 1989). The two adults from New Brunswick were captured in Lindgren funnel traps deployed in an old-growth northern hardwood forest and an old-growth white spruce and balsam fir forest. This species was collected during June and July.

####### Distribution in Canada and Alaska.

AK, YT, AB, MB, QC, **NB** (Bousquet 1989).

**Map 19.**
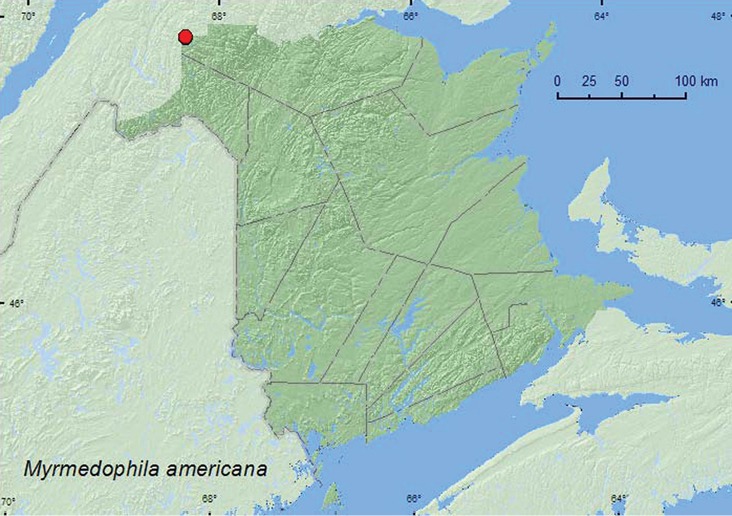
Collection localities in New Brunswick, Canada of *Myrmedophila a*mericana.

###### Pteryngium crenatum

(Gyllenhal, 1808)

http://species-id.net/wiki/Pteryngium_crenatum

Map 20

####### Material examined.

**New Brunswick, Charlotte Co.**, 10 km NW of New River Beach, 45.2110°N, 66.6170°W, 31.V-15.VI.2010, R. Webster & C. MacKay, old growth eastern white cedar forest, Lindgren funnel trap (1, RWC). **Restigouche Co.**, Dionne Brook P.N.A., 47.9064°N, 68.3441°W, 31.V–15.VI.2011, 27.VI–14.VII.2011, 28.VII-8.VIII.2011, 8–23.VIII.2011, M. Roy & V. Webster, old-growth white spruce and balsam fir forest, Lindgren funnel traps (6, RWC); same locality and collectors but 47.9030°N, 68.3503°W, 28.VII–9.VIII.2011, old-growth northern hardwood forest, Lindgren funnel trap (1, RWC). **York Co.**, 15 km W of Tracy off Rt. 645, 45.6848°N, 66.8821°W, 7–14.VII.2009, M.-A. Giguère & R. Webster, old red pine forest, Lindgren funnel trap (1, AFC); 14 km WSW of Tracy, S of Rt. 645, 45.6741°N, 66.8661°W, 10–26.V.2010, R. Webster & C. MacKay, old mixed forest with red and white spruce, red and white pine, balsam fir, eastern white cedar, red maple, and *Populus* sp., Lindgren funnel trap (1, RWC).

####### Collection and habitat data.

This adventive Palaearctic species was reported from bracket fungi in coniferous forests in Nova Scotia by Majka and Langor (2010). The New Brunswick specimens were captured in Lindgren funnel traps deployed in an old eastern white cedar forest, an old-growth red pine forest, an old-growth white spruce and balsam fir forest (boreal forest), an old-growth northern hardwood forest, and an old mixed forest. Adults were captured during May, June, July, and August.

####### Distribution in Canada and Alaska.

BC, QC, **NB**, NS (Bousquet 1991b; Majka and Langor 2010).

**Map 20.**
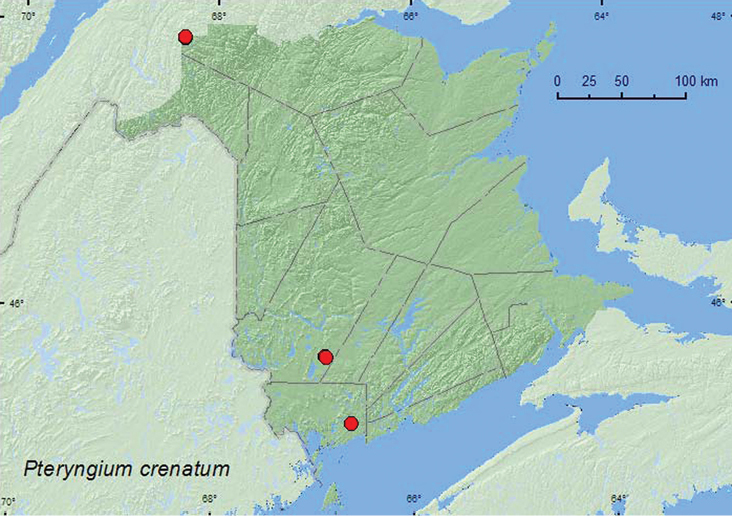
Collection localities in New Brunswick, Canada of *Pteryngium crenatum*.

## Supplementary Material

XML Treatment for Odontosphindus denticollis

XML Treatment for Sphindus species near americanus

XML Treatment for Sphindus trinifer

XML Treatment for Dacne quadrimaculata

XML Treatment for Triplax macra

XML Treatment for Tritoma humeralis

XML Treatment for Tritoma pulchra

XML Treatment for Tritoma sanguinipennis

XML Treatment for Rhizophagus dimidiatus

XML Treatment for Rhizophagus minutus rotundicollis

XML Treatment for Rhizophagus remotus

XML Treatment for Pycnotomina cavicollis

XML Treatment for Antherophagus convexulus

XML Treatment for Cryptophagus acutangulus

XML Treatment for Cryptophagus pilosus

XML Treatment for Cryptophagus mainensis

XML Treatment for Henoticus serratus

XML Treatment for Henotiderus centromaculatus

XML Treatment for Myrmedophila americana

XML Treatment for Pteryngium crenatum

## References

[B1] Bishop DJ Majka CG Bondrup-Nielsen S Peck SB (2009) Deadwood and saproxylic beetle diversity in naturally disturbed and managed spruce forests in Nova Scotia. In: Majka CG Klimaszewski J (Eds). Biodiversity, biosystematics, and ecology of Canadian Coleoptera II. ZooKeys 22: 309–340. doi: 10.3897/zookeys.22.144

[B2] Bouchard P Bousquet Y Davies AE Alonso-Zarazaga MA Lawrence JF Lyal CHC Newton AF Reid CAM Schmitt M Ślipiński SA Smith ABT (2011) Family-group names in Coleoptera (Insecta). ZooKeys 88: 1-972. doi: 10.3897/zookeys.88.807 PMC3088472 21594053

[B3] Bousquet Y (1989) A review of the North American genera of Cryptophaginae (Coleoptera: Cryptophagidae). The Coleopterists Bulletin 43 (1): 1-17.

[B4] Bousquet Y (1990) A review of the North American species of *Rhizophagus* Herbst and revision of the Nearctic members of the subgenus *Anomophagus* Reitter (Coleoptera: Rhizophagidae). The Canadian Entomologist 122: 131-171. doi: 10.4039/Ent122131-1

[B5] Bousquet Y (1991a) Family Rhizophagidae: rhizophagid beetles. In: Bousquet Y (Ed) Checklist of Beetles of Canada and Alaska. Agriculture Canada, Research Branch, Ottawa, Ontario, Publication 1861/E, 218–219.

[B6] Bousquet Y (1991b) Family Cryptophagidae: silken fungus beetles. In: Bousquet Y (Ed) Checklist of Beetles of Canada and Alaska. Agriculture Canada, Research Branch, Ottawa, Ontario, Publication 1861/E, 221–223.

[B7] Bousquet Y (2002) Family 79. Monotomidae Laporte 1840. In: Bousquet Y (Ed) Checklist of Beetles of Canada and Alaska. Agriculture Canada, Research Branch, Ottawa, Ontario, Publication 1861/E, 319–321.

[B8] Bousquet Y Laplante S (2000) Taxonomic review of the Canadian species of the genus *Monotoma* Herbst (Coleoptera: Monotomidae). Proceedings of the Entomological Society of Ontario 130: 67–96.

[B9] Campbell JM (1973) A revision of the genus *Tachinus* (Coleoptera: Staphylinidae) of North and Central America. Memoirs of the Entomological Society of Canada 90: 1-137. doi: 10.4039/entm10590fv

[B10] Campbell JM (1991a) Family Sphindidae: dry-fungus beetles. In: Bousquet Y (Ed) Checklist of Beetles of Canada and Alaska. Agriculture Canada, Research Branch, Ottawa, Ontario, Publication 1861/E, 213.

[B11] Campbell JM (1991b) Family Erotylidae: pleasing fungus beetles. In: Bousquet Y (Ed) Checklist of Beetles of Canada and Alaska. Agriculture Canada, Research Branch, Ottawa, Ontario, Publication 1861/E, 225–226.

[B12] Casey TL (1898) Studies in the Ptinidae, Cioidae and Sphindidae of America. Journal of the New York Entomological Society 6: 61-93.

[B13] Dollin PE Majka CG Diunker PN (2008) Saproxylic beetle (Coleoptera) communities and forest management practices in coniferous stands in southwestern Nova Scotia, Canada. In: Majka CG Klimaszewski J (Eds). Biodiversity, biosystematics, and ecology of Canadian Coleoptera. ZooKeys 2: 291–336. doi: 10.3897/zookeys.2.15

[B14] Downie NM Arnett RH Jr (1996) The beetles of northeastern North America, Volumes 1 and 2. Sandhill Crane Press, Gainesville, Florida, 1721 pp.

[B15] Lafontaine JD Allyson S Behan-Pelletier VM Borkent A Campbell JM Hamilton KGA Martin JEH Masner L (1987) The insects, spiders, and mites of Cape Breton Highlands National Park. Biosystematics Research Report 1. Agriculture Canada, Ontario, 302 pp.

[B16] Lawrence JF Newton AF Jr (1980) Coleoptera associated with the fruiting bodies of slime molds (Myxomycetes). The Coleopterists Bulletin 34: 129-143.

[B17] Leschen RA Skelley PE (2002a) Family 85. Cryptophagidae Kirby 1837. In: Arnett RH Jr Thomas MC Skelley PE Frank JH (Eds) American Beetles. Volume 2. Polyphaga: Scarabaeoidea through Curculionoidea. CRC Press, Boca Raton, Florida, 338–342.

[B18] Leschen RA Skelley PE (2002b) Family 86. Languriidae Wiedeman 1823. In: Arnett RH Jr Thomas MC Skelley PE Frank JH (Eds) American Beetles. Volume 2. Polyphaga: Scarabaeoidea through Curculionoidea. CRC Press, Boca Raton, Florida, 343–347.

[B19] Majka CG (2007) The Erotylidae and Endomychidae (Coleoptera: Cucujoidea) of the Maritime provinces of Canada: new records, zoogeography, and observations on beetle–fungi relationships and forest health. Zootaxa 1546: 39-50.

[B20] Majka CG (2010) The Sphindidae (Coleoptera) of Nova Scotia, Canada. Journal of the Acadian Entomological Society 6: 30-33.

[B21] Majka CG Bousquet Y (2010) Monotomidae (Coleoptera) of the Maritime provinces of Canada. Journal of the Acadian Entomological Society 6: 1-8.

[B22] Majka CG Langor D (2010) Contributions towards an understanding of the Cryptophaginae (Coleoptera, Cryptophagidae) of Atlantic Canada. ZooKeys 35: 13-35. doi: 10.3897/zookeys.35.314

[B23] Majka CG Johnson C Langor DW (2010a) Contributions towards an understanding of the Atomariinae (Coleoptera, Cryptophagidae) of Atlantic Canada. ZooKeys 35: 37-63. doi: 10.3897/zookeys.35.318

[B24] Majka CG Migneault R Webster RP (2010b) *Acropteroxys gracilis* (Newman): the first reports of a lizard beetles (Coleoptera: Erotylidae: Lanuriinae) in the Maritime provinces of Canada. Journal of the Acadian Entomological Society 6: 28-29.

[B25] McHugh JV (2002) Family 75. Sphindidae Jacquelin duVal 1861. In: Arnett RH Jr Thomas MC Skelley PE Frank JH (Eds). American Beetles. Volume 2. Polyphaga: Scarabaeoidea through Curculionidea, CRC Press, Boca Raton, Florida: 305-308.

[B26] Skelley PE Goodrich MA Leschen RAB (1991) Fungal host records for the Erotylidae (Coleoptera: Cucujoidea) of America north of Mexico. Entomological News 102: 57-72.

[B27] Skelley PE McHugh JV (2002) Family 87. Erotylidae Leach 1815. In: Arnett RH Jr Thomas MC Skelley PE Frank JH (Eds). American Beetles. Volume 2. Polyphaga: Scarabaeoidea through Curculionidea, CRC Press, Boca Raton, Florida: 305-308

[B28] Webster RP Klimaszewski J Pelletier G Savard K (2009) New Staphylinidae (Coleoptera) records with new collection data from New Brunswick, Canada. I. Aleocharinae. In: Majka CG Klimaszewski J (Eds). Biodiversity, biosystematics, and ecology of Canadian Coleoptera II. ZooKeys 22: 171–248. doi: 10.3897/zookeys.22.152

[B29] Webster RP Smetana A Sweeney JD DeMerchant I (in press) New Staphylinidae (Coleoptera) records with new collection data from New Brunswick and an addition to the fauna of Quebec: Staphylininae. In: Klimaszewski J, Anderson R (Eds) Biodiversity, Biosystematics and Ecology of Canadian Staphylinidae (Coleoptera) II. ZooKeys. 10.3897/zookeys.186.2469 PMC3349199 22577325

[B30] Woodroffe GE Coombs CW (1961) A revision of the North American *Cryptophagus* Herbst (Coleoptera: Cryptophagidae). Miscellaneous Publications of the Entomological Society of America 2: 179-211.

